# Top journals publishing articles related to drowning prevention: a bibliometric analysis 2000-2022

**DOI:** 10.5249/jivr.v16i1.1840

**Published:** 2024-01

**Authors:** David F Zane, David W Lawrence, Barbara Cosart, Molly B Johnson

**Affiliations:** ^*a*^ Injury Epidemiologist (Ret.), Austin, Texas, USA.; ^*b*^ Member, International Drowning Researchers’ Alliance (IDRA), Kuna, Idaho, USA.; ^*c*^ Director, SafetyLit Foundation, Inc. San Diego, California, USA.; ^*d*^ Trauma Services, Dell Children’s Medical Center, Austin, TX, USA.; ^*e*^ Trauma and Injury Research Center, Dell Children’s Medical Center, Austin, TX, USA.; ^*f*^ Kinesiology Department, University of the Incarnate Word, San Antonio, TX, USA.

**Keywords:** Drowning, Bibliometrics, Periodical, Publishing, Authorship

## Abstract

**Background:**

Drowning is a worldwide problem. Scholarly publications about drowning prevention play a crucial role in bringing data to policy makers and prevention specialists. This study presents a bibliometric analysis of published articles related to unintentional drowning prevention included in the comprehensive, curated injury literature database, SafetyLit®.

**Methods:**

Thorough searches of drowning-related search terms in English and non- English translations identified articles published in journals between 2000 and 2022.

**Results:**

There was a 3-fold increase in publications between 2000 and 2022, with 2,937 articles published in 941 journals. Articles were published in 20 different languages. Five journals published 16% of the articles and sixty-one top journals published 50% of the articles. Eighty-nine percent of the top journals were included in PubMed; 82% were indexed in MEDLINE®; and professional areas of expertise of article authors spanned 18 categories.

**Conclusions:**

This study can facilitate journal selection for drowning researchers to ultimately increase the publication of scientific literature globally.

## Introduction

Drowning is a major public health issue worldwide, resulting in an estimated 236,000 deaths each year and over 2.5 million deaths in the last decade.^1,2^ As the third leading cause of unintentional injury death, drowning accounts for 7% of all injury-related deaths. Children, males, and individuals with increased access to water are most at-risk of drowning.^3^ Although drowning is a significant issue in all countries, drowning rates in low- and middle-income countries are over three times higher than in high-income countries.^3^ Nonfatal drowning further poses a significant public health issue, but the burden is difficult to estimate due to unavailable or unreliable data; lack of drowning data also hinders the ability to evaluate interventions.^1^

Efforts are being made to address the toll fatal and nonfatal drowning has on public health. In 2014, the World Health Organization (WHO) presented current knowledge about drowning and drowning prevention in The Global Report on Drowning: Preventing a Leading Killer, and called for increased efforts and resources to address this preventable public health problem.^3^ In 2017, the WHO provided practical guidance on how to implement 10 effective measures to prevent drowning in Preventing Drowning: An Implementation Guide.^4^ Suggestions included the following interventions: provide safe places away from water for preschool children; install barriers controlling access to water; teach school-age children (aged over 6 years) swimming and water safety skills; build resilience and manage flood risks and other hazards; and set and enforce boating, shipping and ferry regulations. 

It is often unclear what factors drive local and worldwide changes in drowning rates and contexts. Further adding complication to the issue is the fact that drowning intersects with many other topics, such as climate change, infrastructure, migration, and swimming skill development. Each issue is impacted by its own prevention initiatives and policy changes, as well as by political and social climates. Due to this intersectionality, research on drowning prevention may be initiated by experts in a wide range of professional areas of expertise. 

Peer-reviewed publications on drowning provide a critical source of knowledge, which can be used to understand the burden of drowning and the contexts under which drowning occurs locally and globally. Research can also be used to evaluate and guide drowning prevention interventions and to set policy. Between 1995 and 2020, researchers from over 80 countries were engaged in drowning-related research published in peer-reviewed journals.^5^ Additionally, there has been an increase in publications on drowning over time, suggesting a growing level of attention to the critical nature of the problem. We hope to shed light on the need for even more research and publishing to support drowning prevention efforts.

We aim to identify and describe journals that have published peer-reviewed articles related to drowning prevention from 2000 to 2022, to provide data on trends in publishing over time, and to delineate languages journals are published in. We aim to identify top journals, which have published the most articles on drowning. We also aim to identify the professional areas of expertise of authors published by each of the top journals. Our objective is to support the publication and dissemination of scholarly work on drowning prevention for the benefit of the worldwide drowning prevention and research communities. 

## Methods 

**Bibliometric source**

To identify journal articles concerning unintentional drowning, we accessed SafetyLit, a bibliographic database of published scholarly research in the broad field of injury prevention and safety promotion.^6^ SafetyLit is a curated literature database drawing from published research relevant to preventing and researching unintentional injuries, violence, and self-harm. 

SafetyLit provides a comprehensive listing of scholarly items concerning all issues of individual and public safety. Staff and volunteers perform sensitive daily searches of several key databases under license (e.g. PubMed, Global Index Medicus) and text word searches of Google Scholar. Additionally, they perform monthly or quarterly searches of more than 4,000 journals published in 158 nations that are not included in these biomedical databases.^7,8^

SafetyLit records contain bibliographic information, an abstract, a link to the articles digital object identifier (DOI), and a link to the publisher’s website. SafetyLit has a search function with Boolean controls (e.g. AND, OR, NOT) by author, title, journal, year of publication, and text word with optional synonym mapping. Literature records identified can be exported to reference management software.

The SafetyLit database thesaurus^9^ is designed to allow for plain language or technical jargon searches where any query term will simultaneously return all items containing that text-word and all items containing any listed synonym of the word. Thus, a search using the query term “drowning” will also identify all articles that contain phrases such as, “fatal submersion,” “fatal immersion,” “water safety,” “boating safety”. The curation of the SafetyLit system excludes records from the database where the word drowning is used as a metaphor (e.g. “drowning in paperwork”, “drowning in debt”). This thesaurus of synonym terms also facilitates the identification of articles in languages other than English because it can serve as a reference tool for non-English drowning-related terms (الغرق, verdrinking, noyade, Ertrinken, annegamento, غرق شدن, тонущий, ahogo, etc.) for those non-English articles that are without English titles or abstracts. Google Translate was used to translate/transliterate those articles when included in the database.

**Selection criteria**

To identify journal articles and their hosting journal publications relevant to unintentional drowning, we excluded grey literature sources (e.g. technical reports or theses) and focused on articles published between 2000 and 2022. We determined that articles that were exclusively about hospital treatment for nonfatal drowning would be beyond the scope of this project but that articles about treatment that also contained information about drowning risk-factor prevalence, prevention effectiveness, or the individual or societal consequences of fatal or nonfatal drowning, or the costs-of-treatment and rehabilitation would be included. 

To further focus upon recreational or occupational unintentional prevention strategies, we decided to include articles about fatal and nonfatal drownings in bodies of water that are natural (e.g. rivers, lakes, or oceans) and constructed (e.g. swimming pools, bathtubs, buckets, canals, retention ponds, or reservoirs). Articles concerning the forensic determination of drowning intent were included.

There are many definitions of drowning used by authors of journal articles.^10,11,12^ Thus, we did not use our own single definition of drowning in our selection criteria but relied on what the authors themselves had used for their own definitions. If the word “drowning” or one of its synonyms was in the title, abstract, or SafetyLit keyword of the article then that article was initially captured. 

We excluded articles about immersions and suffocations due to incidents such as falls into grain silos or tanks of industrial chemicals. We also excluded articles related to decisions to ignore specific warnings of storms, or to drive or walk into rushing water notwithstanding warning signs. 

**Procedures**

In early March 2023, we performed two Boolean text word+synonym queries of the database. Query 1 ((drown* NOT (abuse OR homicide OR suicide)) identified records containing drowning and synonyms that did not contain words suggesting violence or self-harm. The results of Query 1 were hand-examined to assess relevance and those not related to the prevention or consequences of drowning or nonfatal drowning were excluded. Query 2 ((drown* AND (abuse OR homicide OR suicide)) identified articles that included words suggesting that the record contained information about intentional (suicide or homicide) drownings. These articles were read to identify records that contained information about unintentional drowning in addition to the information about homicidal or suicidal drownings. The articles that also contained information about unintentional drowning were then included. 

Bibliographic information (metadata) on every article (journal, authors, article title, publication year, DOI, language of the journal article) identified by the above searches was saved using the free and open-source Zotero reference management software. A line list of articles was then exported from Zotero to LibreOffice Calc v. 7.3.7.2 (The Document Foundation, Berlin, Germany) and further refined using Microsoft Excel 2011 database functions (Microsoft Corporation, Redmond, WA). 

**Data analysis**

We described the number of journals, number of articles, year of publication, and language of the journal. For this report, we counted conference proceedings published as a journal supplement as one article regardless of the number of abstracts contained in that journal issue. 

All subsequent analyses focused on journals that published eight or more articles related to drowning prevention. We will refer to these as the top journals. We reviewed each article published by the top journals and determined the professional area of expertise of the first and last authors, as indicated by their institutional affiliations and academic degrees. Professional area of expertise categories was determined by author DWL, based on his experience cataloguing research for the SafetyLit database, and were assessed by and agreed to by all authors. 

We also identified if each of the top journals is indexed in MEDLINE, a National Library of Medicine (NLM)® journal citation database; and PubMed, another NLM database of biomedical literature. All items in MEDLINE are included in PubMed.^13^ The PubMed articles are assigned a PubMed identifier (PMID), an essential element of citation references that are included to support assertions made in U.S. government grant proposals.

We further identified the impact factor (IF) for each top journal. IF is one of the metrics used to measure journals' importance in its field.^14^ Impact factors were found in the Clarivate's Web of Science® using the Journal Citation Report tool.^15^ We also determined if the drowning articles had a document online identifier (DOI). A DOI is a unique identifier used to permanently identify a publication and link to it on the internet. Since this was a literature review that involved no human subject involvement or data, no institutional review board approval was sought or required. 

## Results

Between 2000 and 2022, there were 941 journals that published 2,937 articles related to unintentional drowning prevention. 

**Figure 1:**
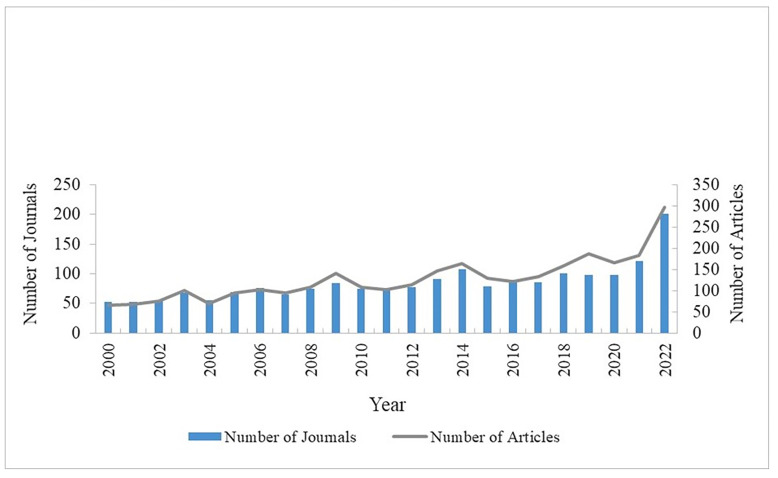
Number of Journals and Articles Published Related to Unintentional Drowning Prevention by Year, 2000-2022.

The number of journals and articles publishing on drowning prevention varied by year (Fig 1). The number of journals with published drowning prevention articles ranged from a low of 53 in 2000 and 2021 to a high of 202 in 2022. The number of articles published per year ranged from 66 in 2000 to a high of 297 in 2022. 

**Journals by language**

**Figure 2:**
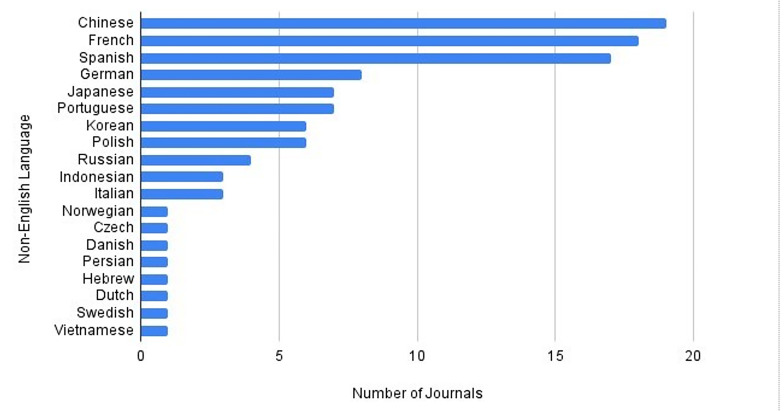
Number of Journals that Published Articles Related to Unintentional Drowning Prevention in Non-English Languages, 2000-2022.

During the study period, we identified articles published in 20 different languages. Of the 941 journals publishing articles related to drowning prevention, 89% (835) were in English. After English, the top three languages journals were published were in Chinese (19), French (18), and Spanish (17), each publishing 2% of the articles. These were followed by German (8), Japanese (7), Portuguese (7), Korean (6), Polish (6), Russian (4), Indonesian (3) Italian (3), and Norwegian, Czech, Danish, Persian, Hebrew, Dutch, Swedish, and Vietnamese (1 each), each publishing 1% or fewer of the articles (Fig. 2).

**Journals publishing the most articles**

Of the 941 journals, the number of articles published in each journal ranged from 1 – 164 (Table 1). Five journals published more than 50 articles each (total of 467 articles). Sixty-one journals published 50% of the articles in our study period. These top journals published between 8 and 164 articles each. 

**Table 1:** Number of Journals by Number of Articles Related to Unintentional Drowning Prevention Published, 2000-2022.

Number of Journals	Number of Articles Published in Each Journal	Total Number of Articles	Cumulative Percentage (and Number) of Journals	Cumulative Percentage (and Number) of Articles
5	>50	467	1% (5)	16% (467)
43	10-50	903	5% (48)	47% (1,370)
13	8-9	109	6% (61)	50% (1,479)
49	5-7	278	12% (110)	60% (1,757)
220	2-4	569	35% (330)	79% (2,326)
611	1	611	100% (941)	100% (2,937)

Table 2 shows the number of articles individually published by the 61 top journals. The five journals that had the most articles published were International Journal of Aquatic Research and Education (164), Injury Prevention (115), Forensic Science International (77), Resuscitation (60), and International Journal of Injury Control and Safety Promotion (51). Eighty-nine percent (54) of the top journals were included in PubMed (Table 2). Seventy-four percent (45) of the top journals had an impact factor; the factor ranged from 0 to 202.731. There was no information on IF identified in online searches for 26% (16) of the top journals. Of the 1,479 articles published in the top journals, 92% had a DOI. We also found that 82% (50) of the journals are indexed in MEDLINE. 

**Table 2:** Top Journals that Published Articles Related to Unintentional Drowning Prevention, 2000-2022.

Journal (Abbreviation)	# of Articles	Medline Indexed	PubMed Included (All Articles)	Impact Factor
International Journal of Aquatic Research and Education (IJARE)	164	N	N	**
Injury Prevention (Inj. Prev.)	115	Y	Y	3.77
Forensic Science International (Forensic Sci. Int.)	77	S1	Y	2.676
Resuscitation	60	Y	Y	6.251
International Journal of Injury Control and Safety Promotion (Int. J. Inj. Control Safe. Promot.)	51	Y	Y	2.603
American Journal of Forensic Medicine and Pathology (Am. J. Forensic Med. Pathol.)	47	Y	Y	1.108
BMC Public Health	46	Y	Y	4.135
Legal Medicine (Tokyo) (Leg.Med (Tokyo))	43	Y	Y	2.017
Journal of Injury and Violence Research (J. Inj. Violence Res.)	39	Y	Y	**
International Journal of Environmental Research and Public Health (IJERPH) 38 Y Y **	38	Y	Y	**
Accident Analysis and Prevention (Accid. Anal. Prev.)	33	Y	Y	6.376
Pediatrics	33	Y	Y	9.703
Journal of Forensic Sciences (J. Forensic Sci.)	29	Y	Y	1.717
American Journal of Emergency Medicine (Am. J. Emerg. Med.)	28	Y	Y	4.093
Journal of Forensic and Legal Medicine (J. Forensic Leg. Med.)	28	Y	Y	1.691
Forensic Science, Medicine, and Pathology (Forensic Sci. Med. Pathol.)	27	Y	Y	2.456
PLoS One	27	Y	Y	3.752
Health Promotion Journal of Australia (Health Promot. J. Austr.)	26	Y	Y	2.033
International Journal of Legal Medicine (Int. J. Legal Med.)	26	Y	Y	2.791
MMWR: Morbidity and Mortality Weekly Report (MMWR Morb. Mortal. Wkly. Rep.)	21	Y	Y	35.301
Fa Yi Xue Za Zhi	20	Y2	Y2	**
Wilderness and Environmental Medicine (Wilderness Environ. Med.)	20	Y	Y	1.479
Diving and Hyperbaric Medicine (Diving Hyperb. Med.)	19	Y	Y	1.228
Journal of Safety Research (J. Saf. Res.)	19	Y	Y	4.264
Zhonghua Liu Xing Bing Xue Za Zhi	19	Y	Y	**
International Maritime Health (Int. Marit. Health)	18	Y	Y	0
Journal of Coastal Research (J. Coast. Res.)	18	N	N	1.11
Pediatric Emergency Care (Pediatr. Emerg. Care)	18	Y	Y	1.602
Journal of Travel Medicine (J. Travel Med.)	17	Y	Y	39.194
BMJ Open	16	Y3	Y	3.006
Aviation, Space, and Environmental Medicine (Aviat. Space Environ. Med.)	15	Y4	Y	**
Journal of Paediatrics and Child Health (J. Paediatr. Child Health)	15	Y	Y	1.929
Medicine, Science, and the Law (Med. Sci. Law)	15	Y	Y	2.051
Natural Hazards (Dordrecht) (Nat. Hazards (Dordrecht))	15	N	N	3.158
Australian and New Zealand Journal of Public Health (Aust. N. Zeal. J. Public Health)	14	Y	Y	3.755
Lancet	14	Y	Y	202.731
Medical Journal of Australia (Med. J. Aust.)	14	Y	Y	12.776
Safety Science (Safety Sci.)	14	Y,5	N	6.392
Archives of Disease in Childhood (Arch. Dis. Child.)	13	Y	Y	4.92
Injury Epidemiology (Inj. Epidemiol.)	13	N	Y	1.769
Archiv Für Kriminologie (Arch. Kriminol.)	12	N6	N6	**
Injury Control and Safety Promotion (Inj. Control Safety Promot.)	12	Y7	Y	**
Injury	11	Y	Y	2.687
Zhonghua Yu Fang Yi Xue Za Zhi	11	Y	Y	**
Acta Paediatrica (Oslo) (Acta Paediatr. (Oslo))	10	Y	Y	4.056
Archives of Pediatrics and Adolescent Medicine (Arch. Pediatr. Adolesc. Med.)	10	Y8	Y	**
Global Health Action (Glob. Health Action)	10	Y	Y	**
International Journal of Circumpolar Health (Int. J. Circumpolar Health.)	10	Y	Y	1.941
Journal of Agromedicine (J. Agromed.)	9	Y	Y9	1.992
Journal of Community Health (J. Community Health)	9	Y	Y	4.371
Journal of Geophysical Research: Oceans	9	N	N	**
Journal of Science and Medicine in Sport (J. Sci. Med. Sport)	9	Y	Y	4.597
Undersea and Hyperbaric Medicine (Undersea Hyperb. Med.)	9	Y	Y	1.143
Archiwum Medycyny Sadowej I Kryminologii (Arch. Med. Sadowej Kryminol.)	8	Y	Y10	**
British Medical Journal: BMJ (Br. Med. J. BMJ)	8	Y	Y	**
Bulletin of the World Health Organization (Bull. World Health Organ.)	8	Y	Y11	13.831
Children (Basel, Switzerland) (Children (Basel))	8	N	Y	2.835
International Journal of Disaster Risk Reduction (Int. J. Disaster Risk Reduct.)	8	N	N	4.842
Journal of the Medical Association of Thailand (J. Med. Assoc. Thai.)	8	N/6	Y	**
Military Medicine (Mil. Med.)	8	Y	Y	1.563
Sudebno-meditsinskaia Ekspertiza (Sud. Med. Ekspert.)	8	Y	Y	**

Note: Top journals are those identified as having published 8 or more articles on drowning prevention between 2000 and 2022 ** No listing in Clarivate Journal Citation Reports (Y=Yes, N= No) 1 "Selectively indexed;" only certain articles from this are indexed in MEDLINE 2 Began in 1997 3 Began in 2014 4 Ended in 2014 continued by Aerospace medicine and human performance 5 The article's open access status and thus database inclusion was supported by the author by paying a large fee 6 Ended in 2017 7 Ended 2004 continued by International journal of injury control and safety promotion 8 Ended 2012 continued by JAMA pediatrics 9 Began in 2003 10 Began in 2002 11 Began in 1965

**Professional areas of expertise by journals**

Table 3 describes the professional area of expertise of the first and last authors that have published drowning-related materials in the top 61 journals identified in this report. The top area of expertise by frequency of journals was sports/recreation/ergonomics/exercise physiology/tourism group. ^17^ Table 3 also identifies which journals published drowning-related articles by the first and last authors’ professional area of expertise. 

**Table 3:** Professional Area of Expertise of Authors by 61 Top Journals that Published Articles Related to Unintentional Drowning Prevention, 2000-2022.

Professional Area of Expertise of Authors	# of Journals	Specific Journals (in abbreviation, and alphabetical order)
Sports/ Recreation/ Ergonomics/ Exercise Physiology/ Tourism	17	Acta Paediatr. (Oslo); Arch. Dis. Child.; Arch. Pediatr. Adolesc. Med.; Diving Hyperb. Med.; Health Promot. J. Austr.; Inj. Control Safety Promot.; Inj. Prev.; IJARE; Int. J. Circumpolar Health.; IJERPH; J. Coast. Res.; J. Saf. Res.; J. Sci. Med. Sport; J. Travel Med.; Med. Sci. Law; Undersea Hyperb. Med.; Wilderness Environ. Med.
Forensics/Law/ Regulations/Policy	14	Am. J. Forensic Med. Pathol.; Arch. Kriminol.; Arch. Med. Sadowej Kryminol.; Aviat. Space Environ. Med.; Fa Yi Xue Za Zhi; Forensic Sci. Int.; Forensic Sci. Med. Pathol.; Int. J. Legal Med.; J. Forensic Leg. Med.; J. Forensic Sci.; J. Inj. Violence Res.; Leg.Med (Tokyo); Med. Sci. Law; Sud. Med. Ekspert.
Injury Prevention and Control	14	Accid. Anal. Prev.; Arch. Pediatr. Adolesc. Med.; Aust. N. Zeal. J. Public Health; Inj. Control Safety Promot.; Inj. Epidemiol.; Inj. Prev.; IJARE; Int. J. Inj. Control Safe. Promot.; J. Inj. Violence Res.; J. Paediatr. Child Health; J. Saf. Res.; J. Travel Med.; Pediatrics; Pediatr. Emerg. Care
Pediatrics (adolescents)	12	Acta Paediatr. (Oslo); Arch. Dis. Child.; Arch. Pediatr. Adolesc. Med.; Aust. N. Zeal. J. Public Health; Children (Basel); Inj. Control Safety Promot.; Inj. Prev.; IJARE; IJERPH; J. Paediatr. Child Health; Pediatrics; Pediatr. Emerg. Care
Pediatrics (infants, young children)	12	Acta Paediatr. (Oslo); Arch. Dis. Child.; Arch. Pediatr. Adolesc. Med.; Aust. N. Zeal. J. Public Health; Children (Basel); Inj. Control Safety Promot.; Inj. Prev.; IJARE; IJERPH; J. Paediatr. Child Health; Pediatrics; Pediatr. Emerg. Care
Work and Occupational Issues	12	Accid. Anal. Prev.; Arch. Kriminol.; Diving Hyperb. Med.; Injury; Int. J. Circumpolar Health.; Int. Marit. Health; J. Agromed.; J. Coast. Res.; J. Saf. Res.; Med. Sci. Law; Mil. Med.; Safety Sci.
Public Health (and surveillance)/ Health promotion	11	Aust. N. Zeal. J. Public Health; BMC public health; Bull. World Health Organ.; Glob. Health Action; Health Promot. J. Austr.; Inj. Control Safety Promot.; Inj. Epidemiol.; Int. J. Inj. Control Safe. Promot.; J. Community Health; J. Inj. Violence Res.; MMWR Morb. Mortal. Wkly. Rep.
Transportation (water)	11	Accid. Anal. Prev.; Arch. Kriminol.; Aviat. Space Environ. Med.; Injury; Inj. Prev.; Int. Marit. Health; J. Agromed.; Journal of geophysical research: oceans; J. Saf. Res.; Safety Sci.; Undersea Hyperb. Med.
Environment (geology, geography, geophysics, engineering)	11	Diving Hyperb. Med.; IJARE; Int. J. Circumpolar Health.; Int. Marit. Health; J. Coast. Res.; Journal of geophysical research: oceans; J. Travel Med.; Nat. Hazards (Dordrecht); PLoS one; Safety Sci.; Wilderness Environ. Med.
Risk Studies (including psychology, psychiatry)/ Risk-taking	10	Acta Paediatr. (Oslo); Am. J. Forensic Med. Pathol.; Arch. Pediatr. Adolesc. Med.; Aviat. Space Environ. Med.; Health Promot. J. Austr.; Int. J. Circumpolar Health.; Int. Marit. Health; J. Travel Med.; Nat. Hazards (Dordrecht); PLoS one.
Medicine (general)	8	Br. Med. J. BMJ; BMJ open; J. Med. Assoc. Thai.; Lancet; Med. J. Aust.; Resuscitation; Zhonghua Liu Xing Bing Xue Za Zhi; Zhonghua Yu Fang Yi Xue Za Zhi
Disasters	7	Injury; Int. J. Disaster Risk Reduct.; IJERPH; Journal of geophysical research: oceans; Nat. Hazards (Dordrecht); PLoS one; Wilderness Environ. Med.
Emergency Medicine and Traumatology	5	Am. J. Emerg. Med.; Injury; J. Inj. Violence Res.; Pediatr. Emerg. Care; Wilderness Environ. Med.
Transportation (land)	5	Accid. Anal. Prev.; Arch. Kriminol.; Injury; J. Saf. Res.; Safety Sci.
Alcohol and Other drugs	4	Accid. Anal. Prev.; Arch. Med. Sadowej Kryminol.; Injury; PLoS one
Elder Adults	2	Inj. Control Safety Promot.; IJARE
Nursing	2	Arch. Dis. Child.; Zhonghua Liu Xing Bing Xue Za Zhi
Young Adults	2	Inj. Control Safety Promot.; IJARE

Note: Table includes only top journals, which published 8 or more drowning prevention-related articles.

## Discussion

We initiated this investigation to identify and describe journals that published articles related to unintentional drowning prevention between 2000 and 2022. We found that there were 941 journals that published 2,937 such articles during this 23-year time frame. We found that publication numbers varied by year, but increased 3-fold between the year 2000 and the year 2022. Approximately one fourth of the journals publishing in 2022 had not published drowning related articles in the previous 22 years. 

A prior analysis of drowning-related articles that limited inclusion to research articles found that the number of published articles grew substantially between 1995 and 2020.^5^ The difference in magnitude of the change they found compared to our findings is likely due to a number of different study parameters (e.g., years range covered, databases searched, selection criteria); both studies are in agreement, however, that publishing is increasing, suggesting change in a positive direction for drowning prevention.

The journals identified in our investigation were published in 20 different languages, 89% in English. A 10-year assessment of drowning articles using a co-word analysis similarly found that more than 80% of articles were in English.^16^ Prior research assessing author affiliations identified 80 different countries had published drowning articles, with the majority of them authored by English-speaking countries: United States, Australia, Canada, and the United Kingdom.^5^ Though English is one of the important languages of science, and may support the availability of information to a worldwide audience, non-English journals containing drowning prevention articles also contribute towards a greater understanding of drowning as a global issue and allow for research to be utilized on a local level. 

In addition to being an issue that affects all countries around the world, drowning is a topic of interest across many intersecting fields. For example, climate disasters increase risk of drowning for people trying to cross flooded roadways and require targeted drowning prevention to encourage safer decisions and support infrastructure that reduces the likelihood of flooding in a particular region or city.^3^ Drowning can be an occupational hazard for the fishing industry.^3^ Drowning can also be linked to infrastructure policy and development if families bathe or wash clothes in natural water sources or if there are no barriers preventing children from accessing bodies of water.^3^ Drowning intersects with many issues of childhood health and well-being, such as the availability of child care or supervision around water.^3^ Additionally, the swimming and water safety proficiency of adults and children and accessibility of instruction links drowning to recreation and sport.^3^

In order to better understand the many academic and occupational fields related to drowning prevention, we identified the professional area of expertise of authors that had published in the 61 top journals. We categorized authors into 18 professional areas of expertise and found that 28% of the top journals had published articles by authors in the most widely published professional area of expertise group ofSports/Recreation/Ergonomics/Exercise Physiology/Tourism. The journals that published the most articles on drowning seemed to draw from a wider array of experts. The International Journal of Aquatic Research and Education published articles by authors from seven of the expertise groups and Injury Prevention had articles by authors from five areas of expertise. Other top journals were more limited in areas of expertise. Forensic Science International only published articles by authors in the area of expertise of Forensics/Law/Regulations/Policy and Resuscitation only published articles by authors in the area of expertise of Medicine (general).

A broad objective of this investigation is to encourage partners in multiple professional areas of expertise to disseminate their scholarly work for the benefit of the worldwide drowning prevention and research community. We hope that the information linking journals and author professional areas of expertise provided in Table 3 can help authors select journals that are best matched to their background so that publishing is not delayed by rejections of articles based on fit, not quality. Among several factors, choosing the best journal that is concordant with the authors’ professional area of expertise can be key to having one’s manuscript accepted and published in a timely manner.

Some authors may use a journal’s impact factor (IF) as a guide to where to publish due to the potentially greater exposure of high IF journals or the positive role publications in high IF journals can play in instances such as evaluations for tenure at academic institutions. Although the use of the IF as a metric of journal quality is controversial, many institutions believe in its value as a measure of the scope of its readership.^17,18^ Niche journals with a narrow focus will always have a lower IF than journals such as JAMA (Journal of the American Medical Association), Lancet, or Science. However, the value of niche journals is that they may reach more of the professionals and practitioners who work in that field.^19^ There can be financial barriers to publication as well. If authors would like their articles to be available to people outside of academic institutions with costly journal subscriptions, they may want to publish in journals that are open access. Yet, many open-access journals charge publishing fees, which can be especially prohibitive for authors from low- and middle-income countries.

Some authors may have limitations on where they can publish. For instance, grant proposals submitted to most U.S. Departments of Health and Human Services (DHHS) agencies require that reference citations include a PMID.^20^ Additionally, the U.S. American College of Surgeons requires Level I trauma centers have at least 10 peer-reviewed articles by their professional staff published in journals included in Index Medicus or PubMed® in a 3-year period.^21^

Even without external limitations guiding journal selection, authors may want the potential searchability and accessibility offered by inclusion of their article in PubMed or MEDLINE. Of the top journals, 89% were included in PubMed and 82% were indexed in MEDLINE, leaving a large proportion of articles not easily found through searches of no-cost biomedical literature databases. Authors may not be aware of key journals publishing on drowning prevention and many relevant articles if they are not retrievable in PubMed or indexed with MeSH subject headings in MEDLINE. 

It is our suggestion that top journals of drowning articles consider applying for inclusion in PubMed or PubMed Central in order to increase their journal's findability and better facilitate the spread of drowning prevention information across the many professional areas of expertise involved. 

Researchers and authors outside the fields of biomedicine may wish to consider publishing in journals included in those biomedical databases. Multidisciplinary databases such as the free SafetyLit, or subscription-based Web of Knowledge®, exist. Literature aggregators, such as EBSCO® and ProQuest® are available at some university libraries and these subscription databases may include niche journals; accessibility to these articles may be limited due to subscription fees or the need to travel to reach the library. 

In this report, we present journal information that might be helpful to those deciding where to publish their drowning-related work. These journal characteristics include the names of the top 61 journals, the number of drowning articles published over a 23-year period in those journals, professional areas of expertise of authors that publish material relevant to drowning by journal, whether the journal is included in PubMed, indexed in MEDLINE, and its IF. Full information about the journals included in this report and their publishers is available.^22^

**Strengths and limitations**

This report is limited by the scope of our article inclusion criteria. We broadly included all journal articles about unintentional drowning that are available in SafetyLit. We did not exclude articles concerning homicidal or suicidal drownings when the article also addressed unintentional incidents. The included articles were not limited to primary research articles or reviews of the drowning literature as in a prior bibliometric analysis.^5^ The articles in this report were published in peer-reviewed journals but we also included important research-letters and editorials from those journals concerning our topic even though these were not necessarily refereed items. Because the publication hurdles are different, we excluded otherwise important grey literature sources such as technical reports on Texas drowning patterns, Australian water safety strategy, and boat injury surveillance.^23,24,25^ Publications written outside of the bounds of standard research or review articles are important to injury prevention fields because it allows for discussion of implications for interventions or policy changes and may go beyond what can be suggested based purely on research results. Our novel taxonomy of authors’ professional area of expertise was subjective. We also chose only to categorize areas of expertise of the first and last authors. 

Similar to the selection criteria in a previous investigation where researchers sought only articles containing the word “drowning”,^5^ we excluded about 60 disaster-related SafetyLit articles about loss of life due to disastrous floods where the author didn’t specifically use the word “drowning” or its synonyms in the title, abstract, and author-provided keywords (see further explanation below). These articles were related to things such as dam or levee failures; unwise behaviors such as choosing to ignore specific storm alerts. These were published in journals that were not among our “top journals” and were journals with a focus on topics such as flood engineering and management, hydrology, and environmental science, and policing. Typically, these were only the single drowning-related article that was published in the journal during our study period. These disaster-prevention-through-engineering journals have articles with an emphasis on infrastructure quality, flaws, and failures. The articles sometimes mentioned “fatalities” or “loss of life” in the body of the article but even these words may not have been used because deaths as a result of these inundation disasters is understood to be a given. The focus of the engineering-articles is on the success or failure of built infrastructure and not the dire consequences of said failure. These journals seem to expect their authors and readers to be engineers and for their authors to write mathematics- and engineering-focused technical reports. Thus, it seemed reasonable for our current article to target only a broader drowning-prevention general audience.

It is possible that the SafetyLit staff and volunteers may have not identified journals and articles that published relevant reports within the study period. Although SafetyLit claims that rigorous attempts were made to perform a comprehensive examination of all the world’s injury-related professional literature, it is likely that some articles (and possibly entire but obscure niche whole journals) were not identified despite thorough search efforts. Potentially there is a wider range of languages publishing on drowning that were not identified with searches biased towards English even if several non-English drowning terms were used.^9^

## Conclusion

This report provides information about journals that publish unintentional drowning prevention-related articles. We found that drowning articles and journals have generally increased since 2000. Spread of drowning-prevention information may be facilitated by expanding searches for articles published in multiple languages and greater inclusion of drowning-prevention journals in PubMed. We found a wide range of professional areas of expertise involved in drowning prevention authorship and suggest efforts be made within publishing and within the professional areas of expertise we identified to highlight the intersectionality of the topic and support cross-pollination of information across disciplines. We hope the information we have provided about the journals publishing articles related to drowning prevention serves as a resource for the global drowning prevention and scientific communities. 

**Acknowledgements**

We thank Alissa Magrum, and Karla Lawson, PhD, MPH, for their contributions to this report.
